# Progress and opportunities for inertial fusion energy in Europe

**DOI:** 10.1098/rsta.2020.0013

**Published:** 2020-10-12

**Authors:** V. T. Tikhonchuk

**Affiliations:** 1 Centre Laser Intenses et Applications, University of Bordeaux – CNRS – CEA, 33405 Talence, France; 2 ELI Beamlines, Institute of Physics, Czech Academy of Sciences, 25241 Dolní Břežany, Czech Republic

**Keywords:** inertial confinement fusion, laser plasma interaction, fusion ignition and burn

## Abstract

In this paper, I consider the motivations, recent results and perspectives for the inertial confinement fusion (ICF) studies in Europe. The European approach is based on the direct drive scheme with a preference for the central ignition boosted by a strong shock. Compared to other schemes, shock ignition offers a higher gain needed for the design of a future commercial reactor and relatively simple and technological targets, but implies a more complicated physics of laser–target interaction, energy transport and ignition. European scientists are studying physics issues of shock ignition schemes related to the target design, laser plasma interaction and implosion by the code developments and conducting experiments in collaboration with US and Japanese physicists, providing access to their installations Omega and Gekko XII. The ICF research in Europe can be further developed only if European scientists acquire their own academic laser research facility specifically dedicated to controlled fusion energy and going beyond ignition to the physical, technical, technological and operational problems related to the future fusion power plant. Recent results show significant progress in our understanding and simulation capabilities of the laser plasma interaction and implosion physics and in our understanding of material behaviour under strong mechanical, thermal and radiation loads. In addition, growing awareness of environmental issues has attracted more public attention to this problem and commissioning at ELI Beamlines the first high-energy laser facility with a high repetition rate opens the opportunity for qualitatively innovative experiments. These achievements are building elements for a new international project for inertial fusion energy in Europe.

This article is part of a discussion meeting issue ‘Prospects for high gain inertial fusion energy (part 1)’.

## 1. Introduction

Sustainable production of large amounts of energy at affordable prices and with a limited effect on the environment is a challenging and unresolved problem. A controversy between the growth of population, increasing inequality in access to natural resources, education and decent living conditions across the world and increasing stress of human activity on the environment and climate can only be resolved by coordinated efforts from all developed countries in improving the energy production and distribution. Development of renewable energy sources and more efficient modes of energy consumption are indispensable elements of the overall energy programme but without sustainable nuclear energy production these measures are incompatible with the growing ecological, economical and political demands.

Nuclear fission is a viable method of massive energy production, but its attractiveness is significantly undermined by the unresolved problems of treatment of radioactive waste, of danger in operating nuclear reactors in near-critical conditions and the high risk of uncontrolled proliferation of nuclear weapons. Nuclear fusion presents evident advantages in all these issues: it does not produce highly radioactive long-living elements but on the contrary, may incinerate them with energetic neutrons. It is also intrinsically stable and the only dangerous element—tritium—can be produced and consumed in place. However, while the fission energy technology has been developed very fast in the 1950s–1960s, the fusion energy has remained at a research level for more than 50 years and prospects for the construction of a commercial fusion reactor and reliable energy production are still undefined.

It is evident that the fusion energy production is a much more complicated process than fission because it requires the maintenance of fuel at extremely high temperatures, but it is also evident that the present scheme of organization of research on inertial fusion energy in the European Union is not sufficiently programme-oriented; it is conducted on a governmental level as basic research without strong contacts to the industry. Scientists are not yet able to propose viable technical solutions attractive for the industrial sector and private companies. In this paper, I consider the progress and difficulties of inertial fusion research in Europe and opportunities that could be realized in fusion science and technology in the near future.

## 2. Background

The major difference between fission and fusion are that the former is initiated by neutral particles—neutrons—there is no electrostatic barrier and a continuous chain of fission reactions can be produced at near equilibrium conditions and at reasonably low temperatures of several hundred degrees Celsius for a long time. The most vulnerable elements exposed to intense neutron irradiation—the fuel rods—can be safely replaced without perturbing the energy production process. By contrast, fusion reactions involve positively charged particles and the necessity to overcome the Coulomb barrier implies that the fuel must be maintained at very high temperatures of several tens of million degrees Celsius in a plasma state without direct contact with any material. This strict condition of high-temperature thermal equilibrium poses strong and as-yet unresolved physics and technical problems.

Two methods of fusion plasma confinement are investigated: magnetic and inertial. Magnetic confinement offers a possibility of continuous quasi-steady reaction, but the available magnetic fields of a few tesla limit the plasma density to such a low value that the minimum plasma volume is about a few hundred cubic metres, implying a large minimum size of energy production unit and a very high construction cost assuming that all technical problems could be resolved [1]. Moreover, a reactor with a large plasma volume and strong magnetic fields poses a large number of secondary problems such as a large tritium inventory, co-existence of hot plasma environment with magnetic coils maintained at cryogenic temperatures and the system of heat and alpha-particles removal from the burning plasma. All these problems will be addressed when the ITER becomes operational in the 2030s.^1^

Inertial fusion operates in a pulsed regime, where the fuel is compressed and heated so fast that a significant fraction of fuel is burnt off during the expansion time [2]. A quantity of energy released in the explosive process is limited by the mechanical, thermal and radiation resistance of chamber walls, so it cannot be more than a few hundred MJ, equivalent to a hundred of kilograms of high explosives. Therefore, the fuel mass in a single ICF pellet is limited to just a few milligrams and compression and burn take place on very small spatial and temporal scales of a few millimetres and several nanoseconds. Potentially, such small size targets may be a base for a compact reactor, assuming the plasma facing materials and driver would be able to withstand the corresponding thermal, mechanical and radiation loads.

Compared to the magnetic fusion reactor that will be operating in a stationary regime with the efficiency defined by a power balance, inertial fusion is a pulsed process and a positive energy balance has to be achieved separately in each explosion. This feature, together with a high reaction temperature, imposes special constraints on the inertial fusion process. The intrinsic energy yield in a fusion of hydrogen isotopes, deuterium and tritium, D + T → He^4^ + n, is a ratio of the total energy that could be released in the fusion reaction, $E_{\text{f}}=\frac{1}{2}N\epsilon_{\text{DT}}$ (where *N* = *N*_D_ + *N*_T_ is the total number of hydrogen ions with *N*_D_ = *N*_T_ and *ϵ*_DT_ = 17.6 MeV is the energy of the fusion products) to the thermal energy of the hydrogen plasma, 3*NT*_ig_ (including electrons) at the temperature *T*_ig_ ≃ 9.5 keV (corresponding to 10% of the maximum reaction rate) needed for ignition of fusion reaction. This intrinsic yield for the DT fusion is about 300, which is a large number by itself but, unfortunately, insufficient for compensating the losses related to incomplete fuel burn (typically 30%), heating and compression efficiency (typically less than 10%), energy conversion efficiency (less than 40% for a thermal process) and the laser driver efficiency (today of a few per cent).

A fusion yield in the inertial process can be significantly increased by using the ‘hot spot’ ignition scheme. In fact, not all fuel needs to be heated to the ignition temperature, but only a small fraction, called the hot spot, which initiates the burn in a cold fuel shell. The energy released in the hot spot should be sufficient to compensate losses and to further increase its temperature and to trigger a burn wave in the cold fuel. This condition provides a criterion on the hot spot areal density and temperature similar to the Lawson criterion in magnetic fusion [2,3]. The remaining fuel needs only to be compressed, which requires much less energy than heating. The time of burning wave propagating into a cold fuel increases with its areal density, so one could burn up to one-third of the fuel loaded in the target.

The hot spot ignition approach is the dominant paradigm of inertial fusion [3,4]. The mainstream conventional scheme consists of achieving both the goals—compression of the fuel and ignition of the hot spot—in a single process by appropriately designing the laser intensity temporal profile and the target structure. Alternative approaches have been also proposed where fuel compression and hot spot ignition are performed with two different laser pulses [5]. They could be more efficient but more demanding in terms of laser power and performance and have not yet been tested experimentally on a real scale because no appropriate laser facility is available.

This short description shows the principal difficulties mounting on the way to inertial fusion. Although no ‘show stopper’ has been identified so far, realization of this approach is extremely challenging in terms of the precision of target fabrication, laser performance and focusing, synchronization of implosion and ignition and energy recuperation. Inertial fusion was the major driver for laser development and enormous progress in laser technology has been made since the invention of the laser 60 years ago. A unique laser facility capable of demonstrating inertial fusion with yield larger than one—National Ignition Facility (NIF)—has already been operating for more than 10 years. However, ignition has not yet been achieved [4] and the laser repetition rate is far below the level needed for sustainable energy production from fusion.

Research in inertial fusion also faces difficulties in funding because of the duality of its applications for simulation of nuclear weapons and energy production. The major research projects and major laser facilities are supported by national defence programmes in the USA, France, UK, China and Russia. Academic research on inertial fusion energy has very limited support; it is not sufficiently coordinated and funded and there are only two multi-beam laser installations—Omega in the USA and Gekko-XII in Japan—of intermediate energy of 30 and 3.5 kJ, respectively. This situation certainly limits our capacity to develop original and efficient fusion schemes and test them in experiments.

Moreover, the existing large-scale laser facilities, NIF in the USA, Laser MegaJoule (LMJ) in France and Shenguang III (SGIII) in China, are designed for the indirect drive implosion and are not well suited for testing direct drive schemes, which are more efficient and better adapted for energy production. In the indirect drive scheme, the laser radiation is transformed in soft X-ray radiation with a near thermal spectrum corresponding to effective temperature of 300–400 eV, which is then used for target ablation and implosion [3]. While X-ray radiation creates a higher ablation pressure and provides a more homogeneous implosion, it implies an efficiency reduction by a factor 5–10 and the increase of mass involved in the process of X-ray generation by a factor of a thousand. Although an inertial fusion reactor for energy production based on the indirect irradiation scheme LIFE [6] has been designed as a part of the NIF project, its viability in terms of efficiency and ecological compatibility is questionable. The project ended in 2014 after NIF failed to demonstrate ignition in the indirect drive scheme.

By contrast, the direct drive approach promises a more efficient use of laser energy and a higher fusion energy gain by applying laser radiation directly on a target [7]. It is better suited for energy production as targets are much lighter but it requires a much better control of homogeneity of laser irradiation. This approach is being developed by scientists from the Laboratory for Laser Energetics (LLE) at the Rochester University hosting Omega and Omega-EP lasers [8].

In addition to these two mainstream ICF schemes there are several alternative approaches. The fast ignition approach aims at the creation of an ignition spark by irradiation of a compressed target by an intense short pulse petawatt laser. It is led by Japanese scientists from the Institute of Laser Engineering at the Osaka University hosting Gekko-XII and LFEX lasers [9]. Another approach is based on a cylindrical implosion of laser-preheated magnetized plasma in a Z-pinch geometry [10]. It is developed by American scientists from the Sandia laboratory and LLE [11].

## 3. Inertial fusion energy research in Europe

Europe has several kJ-class laser facilities in the Czech Republic, Germany, France and UK, which are suited for studying processes of laser-plasma interaction, but do not allow for the performance of implosion and integrated fusion experiments. European scientists have strong experience in international collaboration and pioneered the organization of the first international project dedicated to inertial fusion energy, HiPER [12], but do not have use of a dedicated laser facility. The HiPER consortium brought together 26 laboratories from 10 countries and was supported by the European Research Council. Its mission was to go beyond ignition and provide the scientific, technological and economic basis for construction of a prototype of a commercial inertial fusion reactor. Unfortunately, despite interesting and promising results obtained by the consortium, the project was not extended beyond 2013 because of a lack of national support and competition with a more successful project for the construction of ultra-high intensity lasers: Extreme Laser Infrastructure (ELI). Since that time, only low-level support provided by the EuroFusion consortium and the International Atomic Energy Agency within the Coordinated Research Projects^2^ has maintained the inertial fusion collaboration.

HiPER consortium has selected shock ignition as a baseline scheme for energy production. This choice is motivated by a thoughtful analysis of multiple constraints and conditions. A shock ignition scheme first proposed by Shcherbakov [13] and further advanced by Betti *et al.* [14] is a direct-drive implosion approach using an additional strong shock for boosting the hot spot temperature and facilitating ignition. It allows a more efficient use of laser energy for ablation of the external part of a spherical shell target and compression of the fuel inside. As opposed to the conventional direct-drive scheme, here the shell is imploded at a lower velocity and at a lower entropy in the fuel, thus permitting to achieve higher fuel densities with a lower risk of excitation of the damaging hydrodynamic Rayleigh–Taylor instability when the shell is imploded. The temperature of the hot spot formed in the target centre when the shell collapses is, however, insufficient for ignition. The missed energy is transported to the hot spot with a strong converging shock, which is excited by a special laser pulse-spike, and its propagation is synchronized with the shell implosion. Calculations show [15] that the required laser spike power of 300–500 TW is within reach of present-day high-energy laser facilities, and thus this scheme could be tested in full scale on NIF or LMJ. There is, however, the caveat that these facilities are optimized for indirect drive, paying a significant penalty in drive efficiency and quality of implosion when operated in direct drive.

A target for shock ignition is as simple as a target for the conventional direct-drive implosion. It consists of a double-layer shell filled with a DT gas. The inner shell of a solid deuterium–tritium mixture is covered by an ablator (plastic or carbon). It does not contain other heavy elements such as a gold cylinder (hohlraum) for conversion laser radiation in X-rays in the indirect-drive scheme, or a gold cone for guiding igniting laser pulse in the fast ignition scheme. This is a significant advantage for a power plant’s operation as it produces much less activated debris and high-speed macro-particles that may damage the focusing optics and the reactor first wall. As such, the implosion phase in the shock ignition scheme can benefit from the knowledge already acquired in the conventional direct-drive approach. In this context, LLE scientists recently demonstrated an impressive improvement in direct-drive implosion manifested by tripling the fusion yield in Omega experiments [16].

### (a) Physics of laser plasma interaction under shock ignition conditions

Studies of shock ignition schemes are focused therefore on the characterization of strong shock excitation by an intense laser pulse and its propagation in the target [17]. Laser intensities needed for strong shock creation are one order of magnitude higher than in the conventional direct-drive approach. It is difficult to achieve them in the standard conditions with available laser systems and the physics of laser–plasma interaction under such conditions is largely unexplored. These processes are in the focus of our studies both experimentally and theoretically. In addition to collisional absorption of laser energy in plasma, nonlinear processes are playing an important role. Parametric instabilities, in particular stimulated Brillouin (SBS) and Raman (SRS) scattering and two plasmon decay (TPD), significantly affect the energy balance in the target and produce large amounts of energetic electrons.

Experiments conducted in a planar and spherical geometry demonstrate generation of energetic electrons carrying up to 10–15% of laser energy. They are correlated mainly with SRS excitation and affect the shock strength and amplitude. Depending on their energy, hot electrons may depose energy downstream the shock front and increase its amplitude, or penetrate upstream the shock front, preheat the cold fuel and decrease the shock strength. The first option is beneficial for shock ignition, while the second one is deleterious. For the moment, experiments in a planar geometry did not succeed in generating shock with amplitude larger than 120 Mbar because of limited laser energy and large lateral losses [18]. By contrast, a strong shock excitation has been demonstrated on the Omega facility in a spherical geometry [19]: by using tightly focused laser beams without temporal smoothing the authors succeeded in exciting a shock with amplitude exceeding 300 Mbar on the surface of a solid spherical target. When converging to the centre, it produced pressures largely exceeding a Gbar level. This experiment needs to be extended to a megajoule laser energy. According to theoretical estimates, the shock pressure enhancement is related to the hot electrons generated by SRS and depositing their energy downstream the shock [20]. If synchronized, such a strong shock should be sufficient for hot spot ignition.

The shock ignition approach has required significant improvements in the theoretical model of laser plasma interaction. A description of parametric instabilities and hot electron transport is out of the scope of standard hydrodynamic models of ICF. A full kinetic and electromagnetic description of laser–plasma interaction requires resolution of microscopic spatial and temporal scales, which are incompatible with a macroscopic hydrodynamic model. A simplified treatment of parametric instabilities is possible if the laser intensity in plasma is known. Several methods for evaluation of laser intensity in plasma have been developed accounting for convergence or divergence of neighbouring optical rays [21], representing laser beams as an ensemble of Gaussian (thick) rays [22] or by using an inverse ray-tracing technique [23]. While these techniques are still under development, they are already implemented in three-dimensional hydrodynamic codes and used for interpretation of experiments [24]. The latter approach shows a significant improvement in description of the cross beam energy transfer [25]. Other processes such as temporal and spatial laser beam smoothing in plasma, resonance absorption, excitation of SRS and TPD instabilities and generation of hot electrons can be also accounted for [26]. These developments are important not only for shock ignition but also for all other ICF schemes including direct and indirect drive.

Another important development is related to modelling of energy transport in ICF plasmas. Both electron and radiative transport in fusion plasmas are non-local, they cannot be described in a standard diffusion approximation and more accurate models are needed. The multi-group approach for the photon transport is well-developed and implemented in radiation hydrodynamic codes. The multi-group approach for the electron transport is more complicated as it has to be treated self-consistently with electric and magnetic fields in plasma. An efficient multi-group electron transport model proposed by French scientists [27] has been tested extensively by comparison with several different kinetic Fokker–Planck codes and demonstrated quite good accuracy [28]. It is of interest for both inertial and magnetic confinement fusion. This model is implemented in several radiation hydrodynamic codes in Europe and the USA. It is, however, limited to the cases of thermal transport without magnetic field and does not account for electrons produced in parametric instabilities and resonance absorption. A more general model based on solution of a kinetic equation with a simplified collision integral is under development [29]. Potentially, it can be incorporated in radiation hydrodynamic codes and provide a more general framework for transport of energetic electrons produced by different sources and accounting for self-consistent electric and magnetic fields.

### (b) Physics of inertial fusion beyond ignition

While the major activities related to inertial fusion in Europe are focused on the laser–plasma interaction physics and achieving ignition, studies of reactor physics are also in the scope of our interests. The HiPER project aimed at the demonstration of fusion energy production assuming that ignition will be achieved on NIF shortly. This project gave a strong impulse for the reactor design and material studies for the inertial fusion. A two-step strategy has been proposed: first, construction of an experimental ‘test’ reactor, which will be operating in a safe burst mode of several tens or hundreds of consecutive shots with a low yield and permitting us to test the integration of supplying, control and energy recovery systems and to address the material technology such as final optics, first wall performance and lifetime, tritium breeding, debris handling and target manufacturing. The second step will be construction of a ‘prototype’ power plant for development of a competitive energy production technology.^3^

After the end of the preparatory stage of the HiPER project the work on the reactor design has been stopped, unfortunately, but research on the materials for inertial fusion continues, and it is further supported by the IAEA within the Coordinated Research Projects (see footnote 2). Several important results concerning the plasma facing components, neutron irradiation assessment and protection of final optics have been obtained. As a plasma facing material of the reactor first wall, tungsten has been considered. It has the best proprieties with respect to thermo-mechanical stresses and hydrogen retention. However, it was demonstrated that a coarse-grained tungsten is not sufficiently resistant to the radiation loads [30,31]. It cannot withstand more than 1000 laser shots with a fusion energy release of 250 MJ. Cracks appear at the surface of a sample manifesting fatigue and loss of structural stability. Much more promising properties are demonstrated by a nano-structured tungsten. Its performance has been studied with multi-scale numerical simulations and experiments showing that neutron-induced vacancies are readily attached to the grain boundaries and effectively annealed with interstitials at temperatures about 600 K [31,32].

Another issue of high importance is survival of the final optics, which is directly exposed to the particle and radiation fluxes. Studies of the silica performance under the fast ion irradiation show that swift ions make deep tracks in the material, provoke bond breaking and massive material disorder [33]. No method for mitigation of the ion damage has been proposed so far. A system of electric and magnetic fields protecting the optics from charge particles might be considered. A danger of the neutron direct irradiation of optics consists in creation of colour centres, which absorb laser light and may dramatically reduce the lens transmission. The proposed mitigation method consists of annealing the colour centres by maintaining the optics at a sufficiently high temperature above 800–900 K. However, lenses need to be brought to the working temperature before the reactor operation and temperature homogeneity needs to be maintained with a precision of ±20 K, which presents a serious technical problem [34].

## 4. Perspectives

This short description of ongoing research shows serious and partially unresolved issues on the way to ignition and from demonstration of ignition of fusion reactions to a commercially viable inertial fusion power plant. However, during the last 10 years many interesting and promising results have been obtained: the physics related to laser plasma interactions and target implosion is better modelled and verified in experiments. Significant progress has been made in the material science. However, the scale of activities in Europe relevant to inertial fusion is rapidly decreasing. Fewer people are working in this domain, and fewer papers are published in journals and presented at the conferences. Apparently, the interests of the European community are shifting to more fundamental neighbouring problems of high-energy density science such as laboratory astrophysics, high-field physics and laser-driven particle accelerators.

This decline in the research activities in inertial fusion is a result of the general European politics with respect to the laser research and technology development. Building of multi-petawatt laser facilities and X-ray free electron lasers in several European countries is a strong long-term investment in the fundamental science, but this is also a strong blow to the inertial fusion research. Europe never had any laser facility dedicated to inertial fusion; we do not have any academic laser system with energy larger than 1 kJ and capable of performing implosion experiments. There are only two multi-beam laser systems, Orion in the UK and LMJ in France, but they both are defence-funded with very limited access to the academic community. There is a serious risk that in a few years all academic research in inertial fusion will move outside Europe and the knowledge will be lost.

This situation is, however, in evident contradiction with the growing understanding in society that a safe and abundant nuclear energy and, in particular, fusion energy, is indispensable for sustainable evolution of mankind. It would be a big mistake to invest all funds in magnetic fusion research and abandon all other options of fusion energy. This societal awareness is manifested by the surprising appearance of more than 20 private companies investing in fusion research.^4^ While each of them investigates different paths to fusion energy, the common denominator is the quest for a compact and commercially attractive fusion reactor that can be operational in the next 15–20 years. These companies perform important work by facilitating links between the research organizations and industry and benefit from high level spin-offs offered by the fusion technology development. The first European laser fusion company ‘Marvel Fusion’ was created last year.^5^ It aims to build an experimental laser facility and develop a prototype fusion power plant based on a direct drive implosion and fuel ignition driven by fast ions.

The increasing activity of these private companies demonstrates that the actual level of academic research in inertial fusion energy is insufficient and does not correspond to the needs of society. It is, however, evident that private companies alone are not able to address the enormous complexity of fusion energy technology, which is not only an outstanding technical problem but also an unresolved scientific problem. At the present stage of knowledge, inertial fusion is a valid option, which may provide a technically viable and commercially attractive solution for an efficient and a rather compact reactor, but it needs more public attention and support. A government and private-supported, well-coordinated international programme and a dedicated modern laser facility are needed to boost this research in Europe.

The recent advancements described above and the high level of European scientists involved justify such a coordinated European research programme. Since the failure of the National Ignition Campaign in the USA in 2013, a large number of experiments have been conducted on NIF and other facilities around the world, addressing salient issues of the laser–plasma interaction physics and implosion hydrodynamics. Several alternative implosion schemes for indirect and direct drive have been tested and promising results have been obtained [35]. Significant improvements in the theoretical toolbox and numerical models provide a more accurate and predictable guide in experiments. A bright example of a dynamic coordination between the theory and experiments is a series of integrated experiments in the direct-drive geometry at the Omega facility [16]. The use of an iterative approach between numerical simulations and experiments enabled the improvement of the fuel areal density and the neutron yield by a factor of 3 with the same laser energy. When scaled to the NIF energy, this result corresponds to a higher neutron yield than the one achieved in the best indirect drive shots.

This evident success in understanding of the physics of inertial fusion is accompanied with a significant progress in the target fabrication and laser technology. There are several academic laboratories and private companies in Europe and the UK that are developing technologies for mass target fabrication and delivery and are able to produce rather complicated targets at acceptable prices. Moreover, a new generation of high power lasers operates with pulse energy of a few joules and a repetition rate up to a few Hz, compatible with what is expected in inertial fusion reactors. The next step consists of increasing the laser pulse energy to a kJ level at a high repetition rate. This step will be attained shortly at the ELI Beamline facility, where laser pulses at a kJ energy, ns pulse duration and with a repetition rate of a few minutes will be available for experiments at the end of this year [36]. Such a $\text{kJ}\;{\text{ns}}^{-1}\;\text{module}$ could be a building block for a multi-beam laser facility with total energy of a few hundred kJ fully dedicated to the inertial fusion programme.

Transition to experiments at high repetition rates poses new challenges for diagnostic performance, data storage and manipulation and debris management that have very much in common with the problems of operation of an inertial fusion reactor. Therefore, the scientific and engineering aspects could be addressed jointly and most efficiently within a common international project aiming at commercial energy production and promising many high-level short-time spin-offs.

There are dedicated ICF programmes in the USA and China. Europe is in evident need of such a programme and has a strong scientific and technical background in the domain. That is demonstrated by recent research results and the establishment of private joint ventures in inertial fusion research. However, companies alone cannot shoulder the whole load of ICF research. The private efforts need to be coordinated with a publicly funded ICF research programme in the European Union. Theoretical, experimental and engineering research have to be supported by construction of an ICF-dedicated modern direct drive laser facility capable of testing innovative ideas in physics and technology and technical solutions. Such a facility on the energy scale of a hundred kJ based on state-of-the-art laser technology and current advanced knowledge of laser–plasma and capsule implosion physics can be constructed within the next 10 years and will be the major step on the way to commercial fusion energy production. It will put the European Union at the forefront of research and technology in fusion energy.

## Footnotes

1 See https://www.iter.org/fr.

2 See https://www.iaea.org/newscenter/news/pathways-to-energy-from-inertial-fusion-materials-beyond-ignition-crp-f13016-successfully-completed.

3 See http://www.hiper-laser.org/30aprojectreport.html.

4 See https://www.fusionindustryassociation.org.

5 See https://www.marvelfusion.io.

## Data accessibility

This article does not contain any additional data.

## References

[1] Freidberg J 2007 Plasma physics and fusion energy. New York, NY: Cambridge University Press.

[2] Atzeni S, Meyer-ter-Vehn J 2004 The physics of inertial fusion. Oxford, UK: Clarendon Press.

[3] Lindl JD, Amendt P, Berger RL, Glendinning SG, Glenzer ZH, Haan SW, Kauffman RL, Landen OL, Suter LJ 2004 The physics basis for ignition using indirect-drive targets on the National Ignition Facility. Phys. Plasmas 11, 339–491. (10.1063/1.1578638)

[4] Hurricane OA *et al.* 2014 Fuel gain exceeding unity in an inertially confined fusion implosion. Nature 506, 343–348. (10.1038/nature13008) 24522535

[5] Tabak M, Norreys P, Tikhonchuk VT, Tanaka KA 2014 Alternative ignition schemes in inertial confinement fusion. Nuclear Fusion 54, 054001 (10.1088/0029-5515/54/5/054001)

[6] Dunne M *et al.* 2011 Timely delivery of laser inertial fusion energy (LIFE). Fus. Sci. Technol. 60, 19–27. (10.13182/FST10-316)

[7] Craxton RS *et al.* 2015 Direct-drive inertial confinement fusion: a review. Phys. Plasmas 22, 110501 (10.1063/1.4934714)

[8] Regan SP *et al.* 2019 The national direct-drive inertial confinement fusion program. Nucl. Fusion 59, 032007 (10.1088/1741-4326/aae9b5)

[9] Sakata S *et al.* 2018 Magnetized fast isochoric laser heating for efficient creation of ultra-high-energy-density states. Nat. Commun. 9, 3937 (10.1038/s41467-018-06173-6) 30258053 PMC6158241

[10] Slutz SA, Herrmann MC, Vesey RA, Sefkow AB, Sinars DB, Rovang DC, Peterson KJ, Cuneo ME 2010 Pulsed-power-driven cylindrical liner implosions of laser preheated fuel magnetized with an axial field. Phys. Plasmas 17, 056303 (10.1063/1.3333505)

[11] Barnak DH *et al.* 2017 Laser-driven magnetized liner inertial fusion on OMEGA. Phys. Plasmas 24, 05631 (10.1063/1.4982692)

[12] Edwards CB, Danson CN 2015 Inertial confinement fusion and prospects for power production. High Power Laser Sci. Eng. 3, e4 (10.1017/hpl.2014.51)

[13] Shcherbakov VA 1983 Ignition of a laser-fusion target by a focusing shock wave. Sov. J. Plasma Phys. 9, 240.

[14] Betti R, Zhou CD, Anderson KS, Perkins LJ, Theobald W, Solodov AA 2007 Shock ignition of thermonuclear fuel with high areal density. Phys. Rev. Lett. 98, 155001 (10.1103/PhysRevLett.98.155001) 17501359

[15] Atzeni S, Ribeyre X, Schurtz G, Schmitt AJ, Canaud B, Betti R, Perkins LJ 2014 Shock igntion of thermonuclear fuel: principles and modeling. Nucl. Fusion 54, 054008 (10.1088/0029-5515/54/5/054008)

[16] Gopalaswamy V *et al.* 2019 Tripled yield in direct-drive laser fusion through statistical modelling. Nature 565, 581–586. (10.1038/s41586-019-0877-0) 30700868

[17] Batani D *et al.* 2014 Physics issues for shock ignition. Nucl. Fusion 54, 054009 (10.1088/0029-5515/54/5/054009)

[18] Baton SD, Le Bel E, Brygoo S, Ribeyre X, Rousseaux C, Breil J, Koenig M, Batani D, Raffestin D 2017 Shock generation comparison with planar and hemispherical targets in shock ignition relevant experiment. Phys. Plasmas 24, 092708 (10.1063/1.4989525)

[19] Nora R *et al* 2015 Gigabar spherical shock generation on the OMEGA laser. Phys. Rev. Lett. 114, 045001 (10.1103/PhysRevLett.114.045001) 25679896

[20] Llor Aisa E, Ribeyre X, Duchateau G, Nguyen-Bui T, Tikhonchuk VT, Colaïtis A, Betti R, Bose A, Theobald W 2017 The role of hot electrons in the dynamics of a laser-driven strong converging shock. Phys. Plasmas 24, 112711 (10.1063/1.5003814)

[21] Igumenshchev IV et al. 2010 Crossed-beam energy transfer in implosion experiments on Omega. Phys. Plasmas 17, 122708 (10.1063/1.3532817)

[22] Colaïtis A, Duchateau G, Nicolaï P, Tikhonchuk V 2014 Towards modeling of nonlinear laser-plasma interactions with hydrocodes: The thick-ray approach. Phys. Rev. E 89, 033101 (10.1103/PhysRevE.89.033101) 24730950

[23] Colaïtis A, Palastro JP, Follett RK, Igumenschev IV, Goncharov V 2019 Real and complex valued geometrical optics inverse ray-tracing for inline field calculations. Phys. Plasmas 26, 032301 (10.1063/1.5082951)

[24] Colaïtis A, Follett RK, Palastro JP, Igumenshchev I, Goncharov V 2019 Adaptive inverse ray-tracing for accurate and efficient modeling of cross beam energy transfer in hydrodynamics simulations. Phys. Plasmas 26, 072706 (10.1063/1.5108777)

[25] Turnbull D *et al.* 2020 Impact of the Langdon effect on crossed-beam energy transfer. Nat. Phys. 16, 181–185. (10.1038/s41567-019-0725-z)

[26] Colaïtis A, Duchateau G, Ribeyre X, Maheut Y, Boutoux G, Antonelli L, Nicolaï Ph., Batani D, Tikhonchuk V 2015 Coupled hydrodynamic model for laser-plasma interaction and hot electron generation. Phys. Rev. E 92, 041101 (10.1103/PhysRevE.92.041101) 26565161

[27] Schurtz G, Nicolaï P, Busquet M 2000 A non-local electron conduction model for multidimensional radiation hydrodynamics codes. Phys. Plasmas 7, 4238 (10.1063/1.1289512)

[28] Brodrick JP *et al.* 2017 Testing nonlocal models of electron thermal conduction for magnetic and inertial confinement fusion applications. Phys. Plasmas 24, 092309 (10.1063/1.5001079)

[29] Del Sorbo D, Feugeas J-L, Nicolaï P, Olazabal-Loumé M, Dubroca B, Guisset S, Touati M, Tikhonchuk V 2015 Reduced entropic model for studies of multidimensional nonlocal transport in high-energy-density plasmas. Phys. Plasmas 22, 082706 (10.1063/1.4926824)

[30] Renk TJ, Provencio PP, Tanaka TJ, Blanchard J, Martin CJ, Knowles TR 2012 Survivability of first-wall materials in fusion devices: an experimental study of material exposure to pulsed energetic ions. Fus. Sci. Technol. 61, 57–80. (10.13182/FST12-A13339)

[31] Garoz D, Páramo AR, Rivera A, Perlado JM, González-Arrabal R 2016 Modelling the thermomechanical behaviour of the tungsten first wall in HiPER laser fusion scenarios. Nucl. Fusion 56, 126014 (10.1088/0029-5515/56/12/126014)

[32] Panizo-Laiz M, Díaz-Rodríguez P, Rivera A, Valles G, Martín-Bragado I, Perlado JM, Munnik F, González-Arrabal R 2019 Experimental and computational studies of the influence of grain boundaries and temperature on the radiation-induced damage and hydrogen behavior in tungsten. Nucl. Fusion 59, 086055 (10.1088/1741-4326/ab26e9)

[33] Rivera A, Olivares J, Prada A, Crespillo ML, Caturla MJ, Bringa EM, Perlado JM, Peña-Rodríguez O 2017 Permanent modifications in silica produced by ion-induced high electronic excitation: experiments and atomistic simulations. Sci. Rep. 10, 641 (10.1038/s41598-017-11182-4) PMC5587686 28878323

[34] Páramo AR, Sordo F, Garoz D, Le Garrec B, Perlado JM, Rivera A 2014 Transmission final lenses in the HiPER laser fusion power plant: system design for temperature control. Nucl. Fusion 54, 123019 (10.1088/0029-5515/54/12/123019)

[35] Betti R, Hurricane O 2016 Inertial-confinement fusion with lasers. Nat. Phys. 12, 435–448. (10.1038/nphys3736)

[36] Jourdain N, Chaulagain U, Havlik M, Kramer D, Kumar D, Majerovà I, Tikhonchuk VT, Korn G, Weber S Submitted. The L4n laser beamline of the P3-installation: towards high-repetition rate high-energy density physics at ELI-Beamlines. *Matter Rad. Extremes*.

